# Medical Schools and Medical Education

**Published:** 1905-05-20

**Authors:** 


### MEDICAL SCHOOLS AND MEDICAL EDUCATION.

At the annual meeting of the Medical Society of London, on the 15th instant, the orator, Mr. Henry Morris, F.R.C.S., selected as the subject of his discourse "The Financial Relations between the London Hospitals and their Affiliated Medical Schools, and the Bearing of these Relations upon Medical Education." As an eminent hospital surgeon and distinguished teacher, everything on such topics which falls from Mr. Morris is entitled to full and respectful consideration; but we think it is almost to be regretted that his address was delivered ex cathedra, in the discharge of the duty of his office in the society, instead of at an ordinary meeting, at which it could have been discussed by the members. It would have been highly interesting to learn how far such an audience as the society would furnish would agree with Mr. Morris, and on what points, if any, they would be disposed to differ from him. The main principles which he asserted were that the Medical Education Committee of King Edward's Fund were mistaken in their description of the benefits arising out of the connection between the hospitals and the medical schools as balancing one another; that the separation of anatomical and physiological teaching from the hospitals would be disastrous to everyone concerned, that the so-called preliminary science subjects were threatening to absorb too much of the time of the student and to withdraw him too much from the actual acquirement of medical and surgical knowledge and skill, and they should be taught prior to the commencement of medical education properly so described, and lastly, that the vast extent of London rendered the existence of several medical schools a practical necessity of the situation. By an almost necessary extension of the argument on his last point, he treated the competition of the provincial schools with the London ones as something which has "come to stay," and which no changes in the London methods will be able to prevent. He said that the provincial universities can now impart the necessary learning as well as give the desired degree, and that "the cynics, therefore, should no longer wonder that many students, loving country life and finding near at home the opportunity of getting both the professional training and the university degree, prefer to enter at the provincial universities. Nor is it surprising that their guardians or parents prefer sending them there rather than to London, where they are far away from home influences and surrounded by the distractions and temptations of a great metropolis."

Mr. Morris further urged that, as an essential condition of the supply of trained practitioners for the sick poor throughout the land, it is essential to keep the cost of medical education within the limits of moderate incomes, and he declared that, if the medical schools were by agreement to raise their fees to an amount sufficient to remunerate the teachers adequately and to provide for the expenses of maintenance, only comparatively rich men would be able to enter the profession. In such circumstances, not only would there be an increase in the number of irregular practitioners, but the very poor and the paupers would be unable to obtain medical aid, as a consequence of the reduction of the number of qualified men. The next thing would be an outcry that the poor were suffered to die of curable diseases for want of proper advice and treatment; and the State or the ratepayers would have to subsidise the existing medical schools or to establish new schools of their own. Possibly, said Mr. Morris, the State would have to educate doctors for Poor-law and club practices, just as it provides the professional education of naval and military officers, the training of school teachers, and, quite recently, the instruction in tropical medicine of colonial medical officers.

